# The value of magnetic resonance imaging in the diagnosis of extraspinal osteoarticular brucellosis

**DOI:** 10.1097/MD.0000000000043923

**Published:** 2025-08-15

**Authors:** Chunmei Xiong, Yan Zhao, Mei Han, Jianzhi Li

**Affiliations:** a Department of Radiology, Shandong Provincial Public Health Clinical Center Affiliated to Shandong University, Jinan City, Shandong Province, China.

**Keywords:** brucellosis, diffusion-weighted imaging, extraspinal bone and joint, gadopentetate dextran amine, magnetic resonance imaging

## Abstract

To explore the value of multimodal magnetic resonance imaging (MRI) in diagnosing extraspinal osteoarthritic brucellosis and improve its diagnostic accuracy. Thirty-nine patients diagnosed with brucellosis with corresponding joint pain admitted to our hospital from December 2020 to November 2021 were retrospectively analyzed as study subjects, and all of them underwent MRI, diffusion-weighted imaging, and gadoterate meglumine to observe the MRI imaging features of extraspinal osteoarthritic brucellosis and the affected joints. In order to analyze the diagnostic value of MRI for extraspinal osteoarthritic brucellosis. The shoulder, sacroiliac, and knee joints were the most frequent sites of extraspinal osteoarthritic brucellosis, all accounting for 23.1% of cases, followed by 15.4% of hip joints. The overall lesions showed plaque-like, worm-like, and irregular-shaped damage according to the analysis of the clinical manifestations. Meanwhile, sclerosis at the edges of the lesions was characterized by long T1 and prolonged T2 signal abnormality and high signal in fat suppression-T2-weighted imaging. Overall swelling of the soft tissues in the vicinity of the joint, with long T1 and long T2 signal abnormalities and high signal on fat suppression-T2-weighted imaging. For abscess formation, by analyzing the joint cavity of the patient, the liquid signal shadow of T2 could be seen, and the lesion area showed noticeable uneven sheet enhancement in the enhanced scan. Extraspinal osteoarthritic brucellosis has specific characteristics in MRI, and MRI has significant clinical value in the diagnosis, differentiation, and evaluation of the therapeutic efficacy of this disease.

## 1. Introduction

Brucellosis, also known as Malta fever or undulant fever, is a zoonotic and contagious disease caused by *Brucella abortus*. It is characterized by high morbidity and transmissibility. Various clinical forms of brucellosis arise from infection by this pathogen, with transmission occurring primarily through the digestive tract, respiratory tract, and skin or mucous membranes. Globally, more than 500,000 new cases are reported annually.^[1]^ Patients commonly present with prolonged fever, often accompanied by excessive sweating, musculoskeletal pain, and hepatosplenomegaly. Complications such as arthritis, spondylitis, and osteomyelitis are frequently observed, with joint involvement reported in 25% to 80% of cases.^[2–4]^

In recent years, the incidence of extraspinal osteoarticular brucellosis has been on the rise. Among these cases, arthritis affecting the hip and knee joints is most prevalent. However, clinical manifestations are often nonspecific, and most diagnostic laboratory tests are only available in specialized infectious disease centers, contributing to a reduced detection rate.^[5,6]^ Moreover, the disease often responds poorly to conventional anti-infective therapies and remains moderately contagious. Without timely treatment, patients may develop intravertebral neurological complications or even fatal outcomes.

Imaging modalities such as computed tomography (CT) and magnetic resonance imaging (MRI) are commonly employed for the diagnosis of extraspinal brucellar arthritis. While CT is widely used in clinical settings to assess paravertebral tissues and spinal canals, it offers limited soft tissue resolution and insufficient sensitivity in detecting granulomas and paravertebral abscesses. Additionally, the radiation exposure associated with CT limits its use, especially for repeated evaluations.^[7]^

In contrast, MRI offers superior soft tissue contrast, multi-parametric imaging capabilities, and avoids radiation exposure. In particular, diffusion-weighted imaging (DWI) demonstrates high sensitivity and specificity by reflecting the microstructural characteristics of tissues and the movement of water molecules, making it a valuable tool for early diagnosis and differential diagnosis of brucellosis.^[8]^ A comprehensive understanding of MRI findings in brucellar arthritis is thus essential for early detection, timely intervention, and effective disease control.

MRI can clearly visualize pathological changes in joint ligaments, synovium, and surrounding soft tissues. Brucellar arthritis typically presents as an acute inflammatory condition with synovial thickening, joint effusion, and relatively mild bone destruction. MRI can accurately delineate the extent and severity of joint lesions. In this study, we aimed to evaluate the characteristic MRI features of brucellosis affecting extraspinal bones and joints, with the goal of improving diagnostic accuracy and informing clinical decision-making.

## 2. Materials and methods

### 2.1. Case data

The Ethics Committee approved the present study for Human Studies, Shandong Provincial Public Health Clinical Center, and written informed consent was obtained from all patients (GWLCZXEC2019-98-05). Thirty-nine outpatient and inpatient cases diagnosed with extraspinal osteoarthritic brucellosis from December 2020 to November 2021 were retrospectively analyzed in our hospital. All patients underwent magnetic resonance scanning, and the joints involved included sternoclavicular, shoulder, elbow, wrist, hip, sacroiliac, knee, and ankle. There were 28 males and 11 females aged 22 to 77 years, with a mean age of (51.72 ± 12.79). Most of the patients lived in rural areas and had a history of exposure to cattle, sheep (some of which were diseased or dead lambs or stillborn or aborted sheep), sheep feces, or direct consumption of undercooked beef or lamb. One of the patients had stillborn pigs and sheep in the surrounding area. The analysis was carried out on the clinical manifestations of 39 patients, all of whom had fever, general malaise, loss of appetite, as well as varying degrees of general wandering joint pain, muscle pain, low back pain, leg pain, difficulty in standing and walking, inability to turn over (noticeable after movement and relieved by resting), and swollen and painful testicles. The study was approved and agreed by the Ethics Committee of Shandong Province Public Health Clinical Center and written informed consent was obtained from all patients (GWLCZXEC2019-98-05).

### 2.2. Criteria

1. Diagnostic criteria: refer to WS 269-2019 “Diagnosis of Brucellosis” for relevant content.
2. Epidemiology: prior to the onset of the disease, the patient had contact with livestock with brucellosis infection, or had eaten raw cow and sheep milk and meat products, etc. Or the patient was previously engaged in brucellosis testing, culture, and other related occupations.
3. Clinical symptoms: malaise, joint pain, and persistent fever (including low-grade fever). Some patients had swollen testicles and enlarged livers and lymph nodes. A small number of patients had various types of rashes and jaundice. Patients in an acute and chronic stage might have symptoms such as damage to the bone and joint system.
4. Laboratory examinations involved the selection of pathological specimens from the patient, such as excreta, blood, and bone marrow, followed by culture to isolate *Brucella*. The specimens were subjected to separation and acquisition techniques. Subsequently, the standard agglutination test was conducted, with the titer set at >1:100++. Analysis of the patient’s symptoms was performed, and if the symptoms persisted for more than a year, a titer confirmation of over 1:50++ was typically employed. The anti-human immunoglobulin test (coombs) was initiated with a significant titer of more than 1:400++.

### 2.3. Inclusion and exclusion criteria

Inclusion criteria: a patient with a positive serum *Brucella* agglutination test was diagnosed with extraspinal osteoarticular brucellosis. Patients had corresponding joint symptoms (pain, functional limitation, etc) of the musculoskeletal system. Patients consented to receive imaging examinations and sign the corresponding informed consent form. Patients successfully completed MRI (gadoterate meglumine [GD-DOTA] enhanced scan) examinations. Complete clinical data.

Exclusion criteria: patients might have other diseases causing extraspinal osteoarticular involvement. Patients had a history of surgery or metal implants at the scan site. Patients were unable to complete the MRI examination process. Those with coagulation disorders. Those who have recently received hormonal and anti-inflammatory therapy.

### 2.4. MRI sequence and parameters

A Dutch Philips 3.0T MR (Philips Medical Systems Netherlands Ltd., Eindhoven, Netherlands) instrument performed transverse, coronal, and sagittal scans. Sternoclavicular joint and shoulder joint: abdominal coil and unique coil for the shoulder joint; T1-weighted imaging (T1WI) and proton density-weighted image (PDWI) scans were performed; scan parameters: for T1WI, repetition time (TR)/echo time (TE) results were 473 ms/15 ms, and for PDWI, TR/TE results were 3000 ms/80 ms. Elbow joint: small flexible coil, T1WI, and PDWI scan were performed; scan parameters: for T1WI, TR/TE results were 814 ms/15 ms; for PDWI, TR/TE results were 3600 ms/30 ms. Wrist joint: small flexible coil, T1WI, and T2-weighted imaging (T2WI) scans were performed; scan parameters: for T1WI, TR/TE results were 327 ms/15 ms; for T2WI, TR/TE results were 3600 ms/37 ms. Sacroiliac and hip joints: abdominal coil, T1WI, and T2WI scans were performed; scan parameters: for T1WI, TR/TE results were 633 ms/20 ms; for T2WI, TR/TE results were 3000 ms/60 ms. Knee joints: knee-specific coil, T1WI, and PDWI scans were performed; scan parameters: for T1WI, TR/TE results were 652 ms/20 ms; for PDWI, TR/TE results were 2.6 s/25 ms. Ankle: small soft coil, T1WI, and PDWI scans were performed; scan parameters: for T1WI, TR/TE results were 655 ms/20 ms; for PDWI, TR/TE results were 3400/25 ms. Enhancement scans chose GD-DOTA as the contrast agent, administered at 0.2 mmol/kg.

### 2.5. Processing of images

MRI images were evaluated separately by 2 imaging physicians with 10 years of diagnostic experience and further determined by a superior physician in case of disagreement. Assessment content: assess the involved joints, T1WⅠ/T2WⅠ/short time of inversion recovery bone marrow signals, articular cartilage signals, bone destruction, osseous hyperplasia sclerosis, peripheral soft tissue signals, and abscess formation. The exostoses were evaluated according to the MRI evaluation criteria, with the knee joint evaluation criteria as the main one^[9]^: grade 0: no change; grade I refers to limited low signal and loss of layered structure; grade II cartilage defect depth has not yet reached 50% of the whole layer, and the surface contour shows mild to moderate irregularity; grade III cartilage defect depth is more than 50%; and grade IV cartilage defect of the whole layer.

### 2.6. Statistical analysis

The experimental data were analyzed with IBM SPSS 27.0 (Citrix Marking Software Limited, Suzhou, China), and comparisons between multiple groups were analyzed by one-way ANOVA, with ANOVA chi-square, and the LSD test was chosen. The consistency of judgment between the 2 readers was determined using the Kappa test with k-value results: very low consistency from 0 to 0.2, fair consistency from 0.21 to 0.4, moderate consistency from 0.41 to 0.6, and high degree of consistency from 0.61 to 0.80 and almost perfect consistency from 0.81 to 1.00. Differences were considered statistically significant at *P* < .05. The limitations of the design of this study as a retrospective study prevented some of the key data from being obtained in its entirety, resulting in the failure to calculate diagnostic performance indicators such as sensitivity and specificity.

## 3. Results

### 3.1. Location and number of lessons

**Figure 1.**
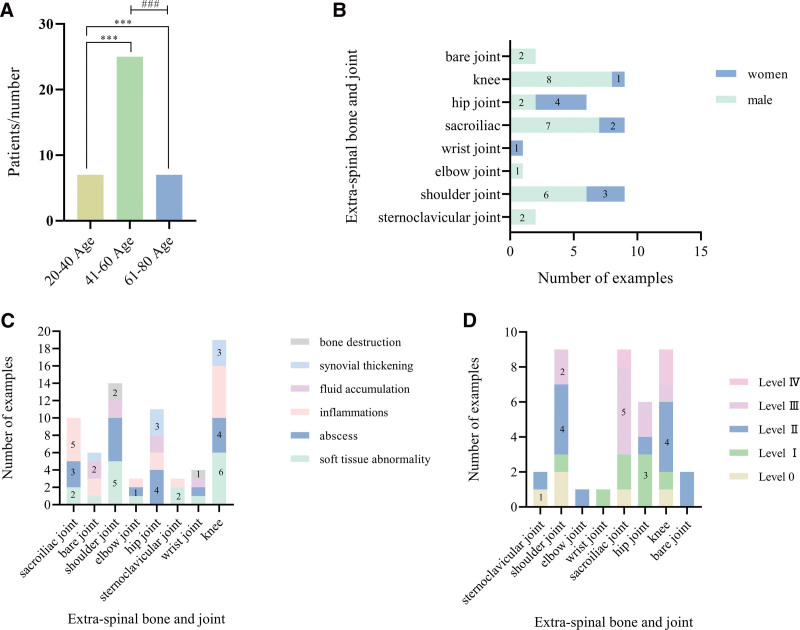
Brucellosis exostosis data charts. (A) Age distribution of 39 patients; (B) distribution of brucellosis exostoses in patients of different genders; (C) distribution of symptoms of brucellosis in different exostoses; (D) graduated presentation of brucellosis in different exostoses. Compared with 20 to 40 years old, *** *P* < .001; compared with 41 to 60 years old, #### *P* < .001.

Among the 39 patients with brucellosis of the extraspinal osteoarthritic joints, the number of cases was 7 in the age group of 20 to 40 years, 25 in the age group of 41 to 60 years, and 7 in the age group of 61 to 80 years (Fig. 1A); the distribution of lesions was as follows (Fig. 1B): 2 sternoclavicular joints (5.1%), 9 shoulder joints (23.1%), 1 elbow joint (2.6%), 1 wrist joint (2.6%), 9 sacroiliac joints (23.1%), 6 hip joints (15.4%), 9 knee joints (23.1%), and 2 ankle joints (5.1%). The aggregate number of affected joints tallied up to 39. This investigation revealed that the shoulder, sacroiliac, and knee joints exhibited the highest incidence of brucellosis in extraspinal osteoarticular joints, with the hip joint following suit.

Among the 8 types of exostoses, soft tissue abnormalities were found in all joints except the hip joint, with the shoulder and knee joints having the highest numbers; symptoms such as abscesses and inflammation were found in most exostoses (Fig. 1C). Among the 39 patients, patients with grade II and grade III injuries predominated, with 13 and 10 patients, respectively, and patients with grade IV injuries had the least number of 3 patients (Fig. 1D).

### 3.2. MRI findings

#### 3.2.1. MRI manifestations

MRI manifestations: Brucellosis invaded the extraspinal bones and joints, which showed worm-eaten, patchy, or irregular-shaped bone destruction of joint constitutive bones, while sclerosis was visible at the edges of some bone destruction; soft tissue around the lesion was swollen, showing cystic, patchy, irregular or grid-like slightly long T1 and T2 abnormal signals, and fat suppression-T2WI (FS-T2WI) showed high signal; the shape of joint gap was irregular, narrow or fuzzy, or even disappear. Some of the synovial membrane was thickened and showed a high signal on FS-T2WI; a long T2 fluid signal was seen in the joint cavity. An enhancement scan with GD-DOTA showed noticeable inhomogeneous patchy enhancement in the constitutive bone and surrounding soft tissue (or combined with ring enhancement, indicating abscess formation). The thick synovial membrane showed noticeable homogeneous enhancement.

#### 3.2.2. MRI manifestations of brucellosis

**Figure 2.**
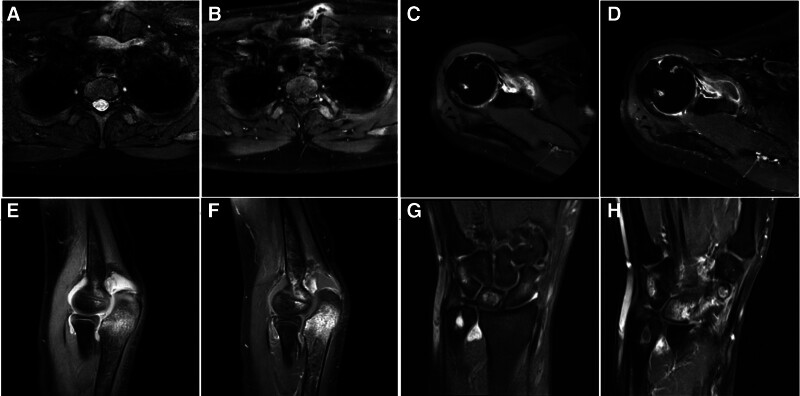
MRI image of brucellosis of the extraosseous joints of the spine. (A and B) MRI images of brucellosis in sternoclavicular joints. (A) The image of the transverse FS-T2WI plain scan. (B) The image of the transverse FS-T1WI enhanced scan. (C and D) MRI images of shoulder brucellosis. (C) The image of the transverse FS-T2WI plain scan. (D) The image of the transverse FS-T1WI enhanced scan. (E and F) MRI images of elbow brucellosis. (E) The coronal FS-T2WI plain scan image. (F) The coronal FS-T1WI enhanced scan image. (G and H) MRI image of brucellosis of the wrist joint. (G) The coronal FS-T2WI plain scan image. (H) The coronal FS-T1WI enhanced scan image. FS-T2WI = fat suppression-T2-weighted imaging, MRI = magnetic resonance imaging, T1WI = T1-weighted imaging, T2WI = T2-weighted imaging.

Examining the sternoclavicular and elbow joints revealed flaky, irregular, slightly prolonged T1 abnormal signals, with high signals in PDWI and FS-T2WI. The interstices of these joints appeared narrow and indistinct, with long T2 liquid signals detected in the elbow joint cavity. Additionally, injection of GD-DOTA resulted in pronounced flaky enhancement of the soft tissues surrounding these joints (Fig. 2A–D). Findings in 9 cases involving the shoulder joint indicated internal bone abnormalities with T1 and T2 signal alterations, space blurring, and bone destruction at the acromion end. FS-T2WI showed high signals in the acromion bone, while long T2 liquid signals were observed in the articular cavity and biceps tendon sheath. Enhanced scans demonstrated enhancement of the joint capsule and tendon sheath (Fig. 2E and F). In a single case concerning the wrist joint, thickening of the peritendinous sheath of the carpal tunnel tendon was noted. Patchy, slightly prolonged T1 and T2 abnormal signals were present in the wrist joint bones and surrounding soft tissues, with high signals in FS-T2WI. Carpal joint space was narrowed and blurry, and enhancement scans exhibited markedly heterogeneous and patchy enhancement suggestive of infection formation (Fig. 2G and H).

**Figure 3.**
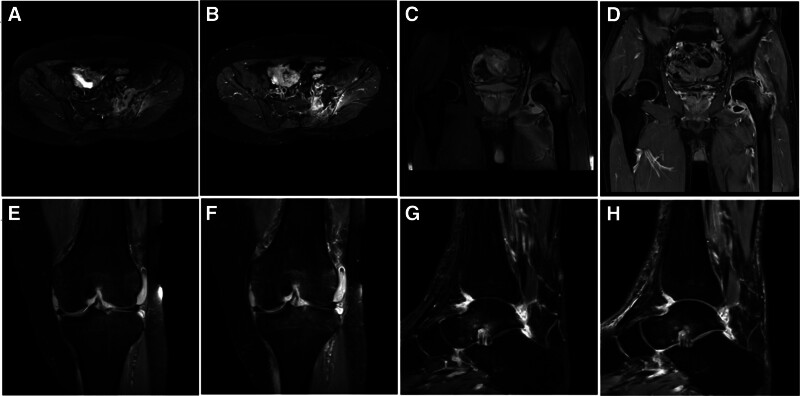
MRI image of brucellosis of the extraosseous joints of the spine. (A and B) MRI images of sacroiliac joint brucellosis. (A) The image of the transverse FS-T1WI plain scan. (B) The image of the transverse FS-T2WI enhanced scan. (C and D) MRI images of hip brucellosis. (C) The coronal FS-T2WI plain scan image. (D) The coronal FS-T1WI enhanced scan image. (E and F) MRI image of brucellosis of the knee joint. (E) The coronal FS-T2WI plain scan image. (F) The coronal FS-T1WI enhanced scan image. (G and H) MRI images of bare joint brucellosis. (G) The sagittal FS-T2WI plain scan image. (H) The sagittal FS-T1WI enhanced scan image. FS-T2WI = fat suppression-T2-weighted imaging, MRI = magnetic resonance imaging, T1WI = T1-weighted imaging, T2WI = T2-weighted imaging.

Nine cases involved the sacroiliac joints, and worm-like, flaky, and irregular bone destruction was seen in the sacroiliac joints, adjacent sacrum, and ilium, with sclerosis of some of the edges of the bone destruction. Overall, slightly long T1 and T2 abnormal signals, high signals in FS-T2WI, irregular joint spaces, blurred articular surfaces, and long T2 fluid signals could be seen in the cavity of the sacroiliac joints. After injection of GD-DOTA, the lesions showed noticeable uneven and ring-like enhancement (Fig. 3A and B). Six cases involved the hip joint, and patchy, slightly long T1 and T2 abnormal signals of different degrees were seen in the acetabulum, femoral head, femoral neck, sciatic branch, and nearby soft tissues, and the synovial membrane of the hip joint was thickened with slightly long T2 abnormal signals. High signals were seen in FS-T2WI, and long T2 fluid signals were seen in the hip cavity. In the enhanced scan images, the acetabulum, femoral head, femoral neck, sciatic branch, and the surrounding soft tissue lesions showed heterogeneous enhancement, and the synovial membrane of the hip joint showed noticeable homogeneous enhancement (Fig. 3C and D).

Nine cases involved the knee joint, knee joint synovial membrane thickening, the surrounding soft tissues visible grid-like and patchy slightly long T1 abnormal signal, popliteal muscle swelling, patchy FS-T2WI high signal, tibialis posterior muscle gap, and the joint cavity can be observed in the long T2 liquid signal shadow. Enhanced scanning showed marked enhancement of the synovium and patchy and grid-like inhomogeneous enhancement of the surrounding soft tissues (Fig. 3E and F). Two cases involved the ankle joint, and patchy and grid-like long T1 and T2 abnormal signals were seen in the lower tibiofibular and talar bones, the heel bone, and the periarticular soft tissues, and patchy FS-T2 WI hyperintense signals were seen in the heel-tarsal ligaments and the heel-fibular ligaments. Also, a long T2 fluid signal was present in the talo-heel and talocalcaneal cavities of the ankle joint (Fig. 3G and H).

#### 3.2.3. Inter-observer consistency test

The Fleiss Kappa test showed inter-observer agreement test K values of 0.79, 0.65, 0.66, 0.65, 0.70, 0.90, and 0.79 for the sternoclavicular, shoulder, elbow, wrist, sacroiliac, hip, knee, and ankle joints, respectively (*P* < .001) (Table 1).

**Table 1:** Observer agreement test for various spinal joint MRI diagnoses.

	Kappa value*	*P*
Sternoclavicular joint	0.79 (0.47–1.10)	.001
Shoulder joint	0.65 (0.32–0.89)	.001
Elbow joint	0.66 (0.37–0.94)	.001
Wrist joint	0.65 (0.34–0.97)	.001
Sacroiliac joint	0.70 (0.42–0.98)	.001
Hip joint	0.90 (0.61–1.18)	.001
Knee	0.79 (0.50–1.07)	.001
Bare joint	0.73 (0.44–1.01)	.001

MRI = magnetic resonance imaging.

* 95% confidence intervals in parentheses.

## 4. Discussion

Brucellosis, a bacterial zoonosis, remains a significant global public health concern, with approximately 1 million documented cases and around half a million new infections reported annually. Spinal involvement is particularly common, occurring in more than half of affected individuals and manifesting as a clinical condition known as brucellar spondylitis.^[10,11]^ The spine is especially susceptible to *Brucella* infection, often resulting in serious and potentially debilitating outcomes.^[12]^ Furthermore, brucellosis poses a substantial economic burden, largely due to its impact on human productivity and livestock health.^[13]^ Among 76 known animal diseases, brucellosis has been identified as one of the most detrimental to disadvantaged populations.^[14]^ Clinically, brucellosis is associated with a spectrum of symptoms, including severe pain, fever, night sweats, fatigue, weight loss, headache, and arthralgia: symptoms that may persist for weeks or even months. In more severe cases, complications such as neurological disorders, endocarditis, orchitis, and bone abscesses may occur.^[15]^ Yin et al^[16]^ reported that approximately 67.92% of patients with brucellar spondylitis exhibit involvement of 2 vertebrae, resulting in limited spinal and joint mobility, persistent pain, and a marked decline in quality of life. Prompt diagnosis and effective treatment are critical to improving outcomes for patients with brucellar spondylitis. Clinical diagnosis relies heavily on both pathological and imaging evaluations. While pathological examination (requiring tissue samples from the lesion) can provide definitive evidence, it is invasive, time-consuming, and impractical for routine or follow-up use in clinical settings.^[17]^ MRI is the preferred diagnostic modality for spondylitis, with a reported 100% detection rate for ankylosing spondylitis. MRI offers multiple imaging sequences with high sensitivity to alterations in tissue water and protein content, enabling detailed assessment of soft tissues and surrounding structures. It allows for accurate visualization of characteristic pathological changes such as granulomas, intervertebral disc destruction, and periosteal reactions.^[18]^

### 4.1. Clinical manifestation

Patients diagnosed with brucellosis typically present with nonspecific symptoms such as generalized weakness, loss of appetite, arthralgia, myalgia, and occasionally the abrupt onset of fever. The most prominent clinical features are persistent fever and joint pain, particularly involving weight-bearing joints, which can lead to significant discomfort, muscle spasms, and restricted mobility. These manifestations are especially common in chronic cases and often lack distinct specificity.^[19]^ Additional clinical signs may include ovarian or testicular involvement, hepatomegaly, splenomegaly, lymphadenopathy, neuralgia, and skin rashes. Diagnostic methods include blood culture, serum agglutination testing with titers >1:160, enzyme-linked immunosorbent assay, and polymerase chain reaction, all of which are instrumental in the accurate identification of *Brucella* infection.^[20]^ Laboratory abnormalities frequently observed in patients may include elevated hematocrit, increased C-reactive protein levels, and decreased hemoglobin concentrations.

### 4.2. MRI manifestation

MRI offers superior sensitivity compared to X-ray and CT in the early detection of abnormal signals within bones, joints, and surrounding soft tissues. However, due to the high signal intensity of bone marrow fat on T2WI, lesions may not be clearly visualized. To address this limitation, fat suppression techniques are often employed in conjunction with MRI to enhance lesion visibility. Bone and joint destruction typically appears as rounded or irregularly shaped areas of bone loss, characterized by long T1 and long T2 signal abnormalities. On FS-T2WI, these lesions generally present as hyperintense signals.^[21]^ Hyperplastic sclerotic margins may form around the lesions, and both chronic and acute lesions may coexist within the same region. Without timely intervention, the infection can progressively involve the entire joint structure, and contrast-enhanced scans often reveal significant enhancement of the affected areas. Destruction of the surrounding soft tissues is commonly observed to varying degrees and may extend into adjacent structures. These changes are typically irregular in shape (capsular, patchy, or reticulated in appearance) with well-defined margins. MRI shows long T1 and long T2 signal abnormalities, with high signal intensity on FS-T2WI.^[22]^ Contrast-enhanced MRI may reveal patchy enhancement or ring-like (cyclic) enhancement, suggestive of abscess formation.

MRI is the only imaging modality capable of directly visualizing the synovial membrane and demonstrates high sensitivity in detecting synovial lesions associated with brucellar arthritis. Synovial involvement typically manifests as mild synovial thickening, prolonged T2 signal abnormalities, and hyperintensity on FS-T2WI. Following administration of gadolinium diethylenetriamine pentaacetic acid contrast, the synovium often exhibits uniform and pronounced enhancement. Previous studies have identified the sacroiliac joint as a common site of *Brucella* involvement beyond the spine, a finding consistent with the observations reported in the present case study.^[23–25]^

### 4.3. Differential diagnosis

Osteoarticular tuberculosis and septic arthritis must be carefully differentiated from brucellosis, as they share numerous clinical and imaging similarities, often leading to a high rate of misdiagnosis.^[26]^ Clinically, all 3 conditions can present with nonspecific symptoms such as fever, excessive sweating, malaise, and diffuse musculoskeletal and large joint pain. Imaging findings also overlap, commonly showing osteoarticular and adjacent soft tissue destruction, narrowing or obliteration of the joint space, and abscess formation, all of which contribute to diagnostic confusion.

Studies have demonstrated that both brucellosis and osteoarticular tuberculosis exhibit imaging features such as bone destruction, osteosclerosis, sequestrum formation, changes in intervertebral space, and soft tissue abnormalities on X-ray, CT, and MRI.^[27]^ Similarly, brucellosis and septic arthritis may present with joint effusion and osseous destruction. Despite these commonalities, several distinguishing features aid in differential diagnosis:

#### 4.3.1. Osteoarticular tuberculosis

This form is typically secondary to pulmonary tuberculosis and is characterized by chronic systemic toxicity, including low-grade afternoon fever, fatigue, and night sweats.^[27]^ Imaging often reveals bone destruction or osteoporosis with minimal osteophytic sclerosis. Cold abscesses with calcification are common, and destruction of cancellous bone with cortical breach is frequently observed. Sequestra are a hallmark finding.^[28,29]^ Tuberculous abscesses tend to be large, sometimes exceeding the size of the primary lesion, and may show distant tracking. MRI with contrast typically reveals thin-walled, smooth abscesses with well-defined borders.

#### 4.3.2. Septic (suppurative) arthritis

Most commonly caused by *Staphylococcus aureus* or *Escherichia coli*, septic arthritis has an acute onset and is marked by high-grade, persistent fever that can exceed 40 °C.^[30]^ It presents with pronounced toxic symptoms and significant neutrophilia.^[31]^ Pain is often severe, and imaging may reveal rapid bone destruction, sequestrum formation, adjacent soft tissue swelling, and abscess development. Appendicular involvement is more common in this form of arthritis.

#### 4.3.3. Osteoarticular brucellosis

Patients often report a history of contact with infected animals or consumption of undercooked beef or lamb. Clinically, it presents with low-grade, fluctuating fever, and typically does not result in sequestrum formation. On MRI, abscesses are usually confined around the affected joints and bones, without evidence of distant tracking or internal calcification, differentiating it from tuberculous abscesses.^[32]^ Brucellar abscesses tend to have thick walls, smaller size, and irregular enhancement, whereas tuberculous abscesses typically have thin walls, larger extent, and smooth enhancement with distant flow extension.^[33,34]^

MRI plays a pivotal role in differentiating osteoarticular brucellosis, tuberculous arthritis, and septic arthritis. These differences arise from their distinct pathophysiological mechanisms. *Brucella* species, as intracellular pathogens, induce granulomatous inflammation, resulting in localized bone destruction and reactive hyperplasia. *Mycobacterium tuberculosis* leads to caseous necrosis and liquefaction, culminating in cold abscess and sequestrum formation. In contrast, pyogenic infections are driven by neutrophilic infiltration and acute osteolysis. Therefore, MRI enables differential diagnosis by assessing the ratio of bone destruction to hyperplasia, the morphological characteristics of abscess walls, and the pattern of soft tissue involvement.

Brucellosis should be considered a differential diagnosis in patients presenting with joint pain, particularly in regions where the disease is endemic or among individuals with occupational exposure. In endemic areas, patients presenting with features of suppurative arthritis should be evaluated for possible brucellosis infection. The diagnostic process requires a multidisciplinary approach, including thorough physical examination, laboratory investigations, and imaging studies. Severe osteoarticular involvement in brucellosis may present as synovitis and bone destruction, which can occur in either unifocal or multifocal patterns. Treatment of osteoarticular brucellosis typically involves a combination of antibiotic therapy and, in some cases, surgical intervention. MRI plays a critical role in joint assessment, offering detailed visualization that can help guide the selection of the most appropriate treatment strategy.^[35–38]^

This study focuses on the analysis of multimodal MRI imaging features of the shoulder, hip, and knee joints to elucidate the characteristic manifestations and distribution patterns of peripheral (extraspinal) osteoarticular brucellosis. By clarifying these multimodal MRI findings, the study aims to facilitate early and accurate diagnosis and provide an important reference for differential diagnosis. However, this study has several limitations. As a retrospective analysis, only the final diagnostic conclusions were documented in the original medical records. The details of independent interpretations during the double-blind review process were not preserved, preventing a standardized assessment of imaging findings. Consequently, sensitivity and specificity could not be calculated, limiting the generalizability of the results. Additionally, the cases analyzed in this study were sporadic and occurred in non-endemic areas, where extraspinal osteoarticular brucellosis is rare. As a result, the sample size was relatively small, and the findings lack the statistical power needed to estimate robust diagnostic performance metrics. Therefore, the conclusions drawn from this study should be considered as a qualitative indication that “MRI demonstrates characteristic features” rather than quantitative evidence of diagnostic accuracy. Future research should adopt a prospective study design with an adequate sample size to allow for the calculation and validation of diagnostic performance indicators such as sensitivity, specificity, and predictive values. Such studies are essential to more accurately define the clinical utility of MRI in the diagnosis of osteoarticular brucellosis.

## 5. Conclusion

In conclusion, multimodal MRI effectively reveals the imaging characteristics of extraspinal osteoarticular brucellosis, including the extent and severity of soft tissue involvement. It serves as a valuable tool for guiding treatment decisions, evaluating therapeutic efficacy, and conducting prognostic follow-up. Nevertheless, this study has several limitations. Due to the relatively low prevalence of brucellar spondylitis in the general population, the sample size was limited, which hindered our ability to perform more detailed subgroup analyses. This constraint may introduce a potential risk of bias in the study findings. Future multicenter studies with larger sample sizes are warranted to enhance the robustness of the results. Additionally, the inclusion of more comprehensive clinical variables for stratified analysis is recommended to further validate the imaging differences identified in this study.

## Acknowledgments

The authors would like to express their gratitude to the Scientific Research and Innovation Cultivation Team of the Shandong Provincial Public Health Clinical Center for their support of this project. We also sincerely thank all the patients who participated in this study. Special thanks are extended to Chuangong Hou, Tingting Dai, Jinhuan Yu, and Jincang Tang for their valuable advice on MRI techniques, as well as to the nursing team in the MRI department for their assistance with contrast agent administration.

## Author contributions

**Conceptualization:** Chunmei Xiong, Jianzhi Li.

**Data curation:** Yan Zhao, Mei Han.

**Formal analysis:** Chunmei Xiong, Mei Han.

**Methodology:** Chunmei Xiong, Yan Zhao.

**Writing – original draft:** Chunmei Xiong.

**Writing – review & editing:** Jianzhi Li.
